# Breaking the cycle: addressing barriers to help-seeking and mental health impacts for male victims of intimate partner violence in low-and middle-income countries

**DOI:** 10.3389/fpubh.2025.1565284

**Published:** 2025-05-06

**Authors:** Miguel Landa-Blanco, Raquel Mejía Sánchez

**Affiliations:** School of Psychological Sciences, Faculty of Social Sciences, National Autonomous University of Honduras, Tegucigalpa, Honduras

**Keywords:** barriers to help-seeking, male mental health, male victims, intimate partner violence, gender norms

## 1 Introduction

Intimate Partner Violence (IPV) is a pervasive public health issue that transcends gender, geography, and socioeconomic boundaries, with significant and far-reaching impacts on the mental and physical health of victims. It profoundly disrupts social dynamics, relationships, and community wellbeing, necessitating a comprehensive and multidisciplinary approach to prevention and intervention efforts. While considerable progress has been made in understanding the role of men as perpetrators of IPV (1), male victims remain an often-overlooked and invisible demographic (2), particularly in low- and middle-income countries (LMICs).

A systematic review and meta-analysis of studies published between 2010 and 2022, encompassing a total of 58,357 male participants, found that 20% of men experience physical violence, 44% psychological violence, and 7% sexual violence perpetrated by their partners (3). Similarly, a study conducted in Brazil examined 1,520 alleged victims of IPV, of whom 222 (14.6%) were men. The study found that 71.2% of violent incidents occurred within the home. Furthermore, the study identified two distinct victim profiles. Group 1 consisted of men over 31 who resided in rural areas, were married, had low educational attainment, and were employed in informal or non-salaried jobs. These individuals predominantly experienced violence within the household, often perpetrated by female partners using weapons, which resulted in soft tissue injuries. Group 2 included men under 31, living in urban areas, who were single, had higher levels of education, and were formally employed. These men typically experienced violence from male ex-partners involving the use of physical force (4). These findings highlight the urgent need to bring greater visibility to IPV experienced by men.

Social stigma and profoundly ingrained gender norms contribute to the revictimization of male survivors of IPV, significantly obstructing their recognition of the need for psychological support. This issue is rooted in structural, cultural, and social factors that sustain barriers to seeking and receiving help (2, 5, 6). In particular, in LMICs, men are often reluctant to seek help due to sociocultural norms that reinforce traditional masculinity, where seeking assistance is perceived as a sign of weakness or emotional vulnerability. As a result, mental health is viewed as a lower priority, further discouraging ictims from reaching out for help. Additionally, economic barriers limit access to psychological support for men in LMICs (7, 8).

This article explores the barriers that male IPV victims face when seeking help in the context of LMICs. By critically examining recent findings and identifying weaknesses in current intervention strategies, we propose culturally sensitive and evidence-based approaches to address these dual challenges. The purpose is to spark constructive dialogue and encourage policymakers and practitioners to adopt more inclusive and practical solutions.

## 2 Mental health impacts of IPV on male victims

Men who are victims of IPV often feel powerless due to the perception that they have no control over the violence they experience. This feeling of incapacity triggers severe mental health consequences, including depression, anxiety, and post-traumatic stress disorder (9). In LMICs, where access to mental health care is often limited, underutilized, and stigmatized, most violence intervention programs are not specifically designed for men to address their unique needs (10, 11), these issues can go unaddressed, exacerbating the long-term consequences of IPV. As a result, the situation becomes amplified, affecting the victim's ability to process trauma and seek solutions for their emotional wellbeing.

The relationship between mental health challenges and help-seeking barriers is bidirectional. On the one hand, the stigma surrounding male victimhood can prevent men from seeking the psychological support they need (10, 12). On the other hand, untreated mental health issues can reinforce feelings of isolation and helplessness, creating a cycle of neglect and harm. Without access to appropriate care, male IPV victims may resort to maladaptive coping mechanisms, such as substance abuse or withdrawal from social relationships (9, 13), further compounding their struggles.

## 3 Barriers to help-seeking for male IPV victims in LMICs

### 3.1 Stigma and gender norms

In many LMICs, deeply rooted cultural and societal norms uphold rigid notions of masculinity (14). For instance, in settings where machismo is normalized, men are often expected to embody strength, resilience, and emotional stoicism rigidly. However, traditional elements of machismo, like dominance, sexism, and emotional restraint, were linked to anxiety, especially in the form of cynical hostility, characterized by negative attitudes toward others and a deep mistrust of people (15, 16). Such traits make it difficult for male IPV victims to acknowledge or report their experiences (17). Admitting to being a victim of IPV can be perceived as a failure to conform to these ideals, leading to shame and fear of ridicule.

Studies have consistently highlighted stigma as a significant deterrent to help-seeking among male IPV victims. Men who disclosed experiences of IPV were often met with disbelief and scorn from peers and community members (18–20). In this regard, male victims of IPV by female perpetrators are frequently represented by the media as illegitimate victims, with victim-blaming narratives prevailing. These portrayals often depict them as failures in roles such as fathers, providers, or husbands or as individuals characterized by substance abuse or violent behavior (21). This stigma not only perpetuates harmful gender norms but also reinforces the barriers that prevent male victims of IPV from seeking the help they need (22). Confronting these cultural and societal constructs is essential for creating a more inclusive and supportive environment for all survivors of IPV. Additionally, it is crucial to understand the violent dynamics of each environment and the specific needs of each group, ensuring that no person is stigmatized or labeled. This approach fosters a more empathetic and effective response to IPV survivors, promoting their healing and social integration without reinforcing harmful stereotypes or biases.

### 3.2 Lack of resources and inclusive services

Another significant barrier is the lack of men-inclusive IPV support services (13, 23), something even more prevalent in LMICs. Many programs and interventions are designed with female victims in mind, leaving male survivors with few, if any, options for assistance. Shelters, hotlines, and counseling services that cater specifically to men are rare, and those that exist are often underfunded and inaccessible (6, 24, 25). In addition, healthcare providers and law enforcement personnel in LMICs may lack training on how to recognize and address IPV in general (26), and particularly against men (12, 27). This lack of awareness can lead to dismissive attitudes or inadequate responses, further alienating male victims. Legal frameworks in many LMICs fail to address IPV against men adequately. Gender-specific definitions of domestic violence often exclude male victims, limiting their ability to seek legal recourse (28). Furthermore, societal biases may influence the enforcement of existing laws, with male victims facing additional hurdles in being taken seriously by the judicial system. Addressing these gaps requires not only legislative reform but also a shift in cultural attitudes toward gender and victimhood.

## 4 Strategies for intervention

Interventions for victims of IPV require strategies tailored to their unique needs and the barriers they face in seeking help. Public education initiatives are essential for challenging harmful stereotypes and normalizing help-seeking among male IPV victims. These campaigns should be tailored to the cultural contexts of LMICs, leveraging local traditions and community leaders to promote positive change. Violence prevention programs should focus on emotional regulation and developing interpersonal skills. Psychoeducational components play a critical role by providing participants with knowledge about their emotions and behaviors A key element in these programs is fostering empathy and validation, as these skills enhance recognizing and accepting one's feelings and those of others (29). Additionally, the presence of qualified educators in violence prevention programs is essential to effectively address and mitigate the impacts of IPV. Educators must possess a deep understanding of the dynamics of IPV, including its social and cultural dimensions, and be capable of conveying complex information with empathy in order to engage participants meaningfully. Furthermore, they should actively promote healthy relationships and their core attributes, such as decision-making and non-violent conflict resolution (30, 31).

As well, integrating mental health services into primary healthcare systems is a practical way to reach underserved populations in LMICs. Task-shifting—where non-specialist providers are trained to deliver basic psychological support—has shown promise in addressing mental health needs in resource-limited settings (32). Training programs in health services have been shown to enhance confidence and preparedness in addressing the needs of male IPV victims, thereby facilitating better identification, support, and referral to specialized services (6, 33).

Creating gender-inclusive support systems is essential for addressing the needs of male survivors of IPV. Shelters, hotlines, and legal aid services should be designed to include men, providing them with a safe and supportive environment. Partnerships with community-based organizations can foster trust and ensure these services are accessible and culturally appropriate. Furthermore, developing specialized services that address the specific needs of male victims acknowledges their unique experiences and challenges, enabling more comprehensive care. This approach is crucial for dismantling stereotypes and improving support systems (34–36). Similarly, technology plays a fundamental role in providing support to victims of IPV by offering accessible interventions. Digital tools offer simple, accessible, discreet, and non-judgmental means through which men can more easily seek help (37, 38). For male IPV victims, these services should include trauma-informed care and counseling tailored to their unique experiences. Mobile health platforms can also be critical in expanding access, offering anonymous and culturally sensitive support to individuals reluctant to seek help in person (39, 40).

## 5 Discussion

Despite increasing recognition of IPV as a significant public health concern, male victims in LMICs remain largely overlooked and underserved. This marginalization is driven by societal attitudes, limited resources, and policy gaps that reinforce stigma and create barriers to accessing support services. Breaking the silence surrounding IPV experienced by men requires a multifaceted approach that prioritizes inclusivity, cultural sensitivity, and evidence-based interventions to address their unique needs and foster equitable support systems.

IPV is a significant public health issue with profound implications for victims' mental and physical wellbeing. The severity of IPV's impact extends beyond gender and borders, influencing victims across different social, cultural, and economic contexts. While substantial research has focused on male perpetrators of IPV, the experiences of male victims remain largely underexplored, particularly in LMICs. These countries, where access to mental health care is often limited and stigmatized, represent a unique challenge in addressing IPV among men. This discussion explore the barriers male victims of IPV face when seeking help in LMICs, analyze their implications, and propose solutions to better support this often-overlooked demographic.

Gender roles and machismo, where vulnerability is not typically associated with men, complicate the recognition of male victimization, leading to severe mental health impacts. The interplay between barriers to help-seeking and mental health consequences creates a cycle of neglect, perpetuating suffering and undermining public health efforts. By addressing stigma, expanding access to mental health resources, and developing inclusive support systems, we can break this cycle and foster a more equitable and compassionate response to IPV. These efforts must be integrated within communities to reshape perceptions of masculinity, normalizing the idea that men can seek help without fear of judgment. Additionally, prevention and education programs should be integrated with community leaders, as they can aid in normalizing the experience of male victims, thereby contributing to the creation of a more supportive environment for all IPV survivors.

Cultural norms and local traditions significantly shape the perception of men as victims of IPV. In this context, raising awareness through mobile applications, digital helplines, and social media in LMICs can provide accessible and anonymous support. These tools have the potential to foster an inclusive narrative and contribute to normalizing help-seeking behavior among men. Likewise, these tools can bridge the gap between awareness and action, offering psychological resources or counseling that are free from labels and confidential. By utilizing these platforms, it is also possible to identify isolated populations and recruit them for help-seeking due to cultural stigma and geographic or economic barriers.

Collaboration between researchers, policymakers, and practitioners is essential for sustainable change. By fostering open dialogue and sharing best practices, stakeholders can develop innovative strategies aimed at raising awareness and improving accessibility through digital support platforms and psychoeducational resources on social media, as well as qualified educators and frontline support with a deep understanding of the issue, cultural sensitivity, and the ability to communicate with different community groups to intervene and educate. This approach addresses the barriers and mental health impacts faced by male IPV victims in LMICs. Current research on male IPV victims in LMICs is limited, leaving significant gaps in understanding the scope of the problem and the most effective solutions. Future studies should focus on disaggregating data by gender to better capture the experiences of male survivors and evaluate the impact of interventions specifically designed for this population.

It is time to move beyond gender stereotypes and recognize the diverse realities of IPV. Only through inclusive and culturally sensitive approaches can we ensure that all survivors, regardless of gender, receive the support they need to heal and thrive. This requires a social commitment to restructuring attitudes, the development of integrative and accessible support systems, as well as greater community involvement in creating empathetic environments for survivors. By fostering understanding and awareness, we can empower victims to seek help without fear of stigmatization, ultimately promoting the psychological and social wellbeing of all IPV.
